# Using (disruptive) eco-visualization to re-connect humans to nature: results from workshops with youth and adults

**DOI:** 10.3389/fpsyg.2025.1690875

**Published:** 2025-11-04

**Authors:** Erica Löfström, Chiara Santandrea, Christina Carrozzo Hellevik

**Affiliations:** ^1^Department of Psychology and Department of Design, NTNU – Norwegian University of Science and Technology, Trondheim, Norway; ^2^Norwegian University of Science and Technology – NTNU, Trondheim, Norway; ^3^Department of International Business, Norwegian University of Science and Technology – NTNU, Ålesund, Norway

**Keywords:** eco-visualization, art, creativity, eco-anxiety, nature-connectedness

## Abstract

In the Anthropocene epoch, opportunities for nature connectedness are diminishing, raising concerns for both individual well-being and the nurturing of environmental mindfulness in upcoming generations. The prevailing discussion emphasizes the urgency of strengthening our connection with nature, but this viewpoint still largely treats nature as a resource for human benefit. We believe that this human-centered perspective needs re-evaluation, and that a major shift of our understanding of the earth’s ecosystems and our role in it may be necessary. In this study we bring together the latest environmental science assessments on planetary health with eco-critical research and the field of design. We explore how non-technological and technological interventions can facilitate a reconnection with nature. With the ambition to not only evoke emotional resonance with nature, but to also use this as a starting point for collective reflections and joint co-creation of a more sustainable future, we endeavor into a transformative approach. As a first step, we carried out two explorative reflection workshops with different stakeholders: one in a rural setting and one in an urban one, using transformative experiences (disruptive eco-visualizations) as interventions with the intent to evoke an emotional response amongst the workshop participants. This emotional response was used as a starting point for reflections on our current and potential future role as humans in the eco-system. The results show that the participants in the first workshop achieved a reconnection with nature, but some also showed signs of biophobia. In the second workshop, the young participants showed a very cynical view of the future of humanity through the use of art and sense of humor. These results point to the fact that it is more urgent than ever to find ways to reconnect people with nature, especially young adults, to counter the effects of eco-anxiety.

## Introduction/background: the disconnect

The United Nations Climate Panel has repeatedly and unequivocally signaled ‘code red for humanity’, highlighting the urgent juncture at which we find ourselves regarding climate change and environmental degradation (IPCC, 2021; Brondizio et al., 2019). Despite the severity of these proclamations, substantial large-scale actions remain largely elusive. One explanation may be that we are ‘disconnected’ from nature and therefore do not think of ourselves as part of an ecosystem (Beery et al., 2023). Nature disconnection has been defined in the literature as the lack of awareness or disregard for human identity in material elements and within flows, energy and other nonmaterial elements and values that constitute nature (Beery et al., 2023).

Whilst climate change and the loss of biodiversity continue to worsen unabated, it is getting harder to find chances to connect with nature, at least in cities, where 4 billion of the global population lives – (United Nations Population Division, via World Bank, 2025; Soga and Gaston, 2016; Gaston and Soga, 2020). The ongoing alienation of humans from nature driven by urbanization, termed ‘extinction of experience’ (Pyle, 1993), not only entails a loss of personal benefits from being in nature, but also leads to a cycle of disaffection that can have disastrous consequences. Pyle emphasized that direct, personal contact with natural environments is vital in forging a person’s emotional intimacy with nature and cannot be replaced by vicarious experiences (Pyle, 1993). Nature interactions are defined as individual interactions where a person resides in the same physical space as nature or perceives it through a stimulus (Soga and Gaston, 2016). These interactions are considered rich in their ability to engage multiple senses, notably smell, touch and hearing that are richer than interactions in human-built contexts (Kaplan and Kaplan, 1989).

Another effect of urbanization and our growing disconnection from nature is that we lose the perspective of the impacts of our consumption (Sussman, 2022). Indeed, in cities, natural resource extraction is out of sight and out of mind in a society disconnected from nature and fueled by hyper-consumerism of short-lifespan or single-use objects. Our societies disconnected from nature, but paradoxically hyper-connected to other human societies, can cause anxiety (Bear et al., 2025). This is exacerbated by our growing awareness to and the daily evidence of climate collapse around the globe (Brophy et al., 2023). Worryingly, studies show that young people today are anxious about the future and do not feel much hope about our ability to stop climate change. For example, Hickman et al. (2021) carried out a survey with more than 10,000 children and young people (16–25 years) and concluded:

“*Climate anxiety and dissatisfaction with government responses are widespread in children and young people in countries across the world and impact their daily functioning. A perceived failure by governments to respond to the climate crisis is associated with increased distress.*”

Hickman et al. (2021, p. 863)

This stresses the urgency to promote a deeper, experientially grounded connection to our natural surroundings. A reconceptualization is in order, one that fosters a collective emotional symbiosis with the biosphere and ecosystems and awareness of our environmental impacts. In this paper, we explore a playful approach to reconnect with nature, using eco-visualization, art and creativity to spark new ways of approaching nature. We analyze the responses at two workshops carried out in Summer 2023, the first in Denmark, and the second in Norway. We then draw conclusions tying the findings to existing literature on climate and eco-anxiety, suggesting ways of changing paradigms to reconnect to nature and start the healing process of the biosphere and our own psyche.

## Research questions

A common challenge in sustainability and biodiversity conservation is that people struggle to relate to what is beyond them and other humans, in other words, the more-than-human world (Droz, 2022). This reinforces the *anthropocentric* view of the world (Woodhall, 2017), namely the understanding and interpretation of the world in terms of human values and experiences (Talgorn and Ullerup, 2023). The focus on human needs alone reinforces unsustainable development and further disconnection from nature, as a vicious cycle (Soga et al., 2023); whereas the consideration of human and non-human needs is necessary to design solutions that are beneficial to all stakeholders including nature. By initiating reflection workshops from this vantage point, we aspire to foster a more harmonious coexistence with the natural world, engendering collective strategies and actions that are informed by a deep-seated, genuine affinity and respect for the web of life that constitutes our biosphere; a system that we humans have at least partly disconnected from as a species through a process of ‘bifurcation’ (Beery et al., 2023). This is the starting point to explore whether a nature-centered or ecocentric approach – rooted in direct experiences and emotional connectedness – can forge a fertile ground for collective deliberations and actions aimed at restoring and maintaining a harmonious equilibrium with the natural environment.

With the aim to convey ecological insights in a manner that engages senses and emotions, we used different transformative experiences – disruptive eco-visualizations (Löfström and Fjællingsdal, 2022). Disruptive eco-visualizations belong to a novel approach, Disruptive Environmental Communication, which differs from other environmental communication approaches in that the intent is to provoke an emotional response rather than simply to convey neutral facts (Klöckner and Löfström, 2022). In our study we use disruptive eco-visualizations as interventions that form the basis for further reflections amongst the workshop participants on our current and potential future role as humans in the ecosystem. Our research questions are:

- How can we convey ecological insights in a manner that resonates not merely with the cognitive faculties but profoundly engages emotions?
- How can we use these insights as a starting point for collective reflections and co-creation of a more nature-connected lifestyle?

In order to answer these questions, we will now explore some theoretical concepts that will help us navigate existing theory with the new approach we propose.

## Theory

In this study, we advocate for a paradigm shift towards a more encompassing, ecocentric focal point, harnessing these grounded experiences not merely as avenues for personal enrichment, but as catalysts to spur dialogues grounded in a holistic ecological consciousness. Hence, the visualizations used in our study were designed or chosen because they, contain elements that in different ways challenges human exceptionalism. This altered perspective seeks to facilitate discourses and reflective processes that gravitate beyond the human-centered narrative, exploring the viability of nurturing empathy towards nature at large (Pascual et al., 2023). To hone such a shift, it is necessary to understand specifically what the challenges are.

### Breaking the boundaries

We currently live in the so-called *Anthropocene Epoch* (Rockström et al., 2009), marked by the dominant influence of humans on the Earth System (Crutzen, 2002). This is manifested in the large-scale land-use change, climate change, natural resource depletion and the discharge of chemical pollutants, or ‘novel entities’ (Rockström et al., 2009). Land-use change in the form of urbanization profoundly impacts the landscape through habitat loss and fragmentation which present a significant threat to biodiversity and ecosystem function because it exploits and changes the balance and relationship of the different species that live in an area (Goddard et al., 2010; Campbell-Arvai, 2019; Seto et al., 2012).

In 2023 (Richardson et al., 2023), a team of researchers made the first-ever quantification of all nine processes governing the Earth’s stability and resilience. The framework known as Planetary Boundaries was first defined by Rockström et al. (2009), updated by Steffen et al. (2015) and revised several times for individual boundaries (e.g., Villarrubia-Gómez et al., 2018; Wang-Erlandsson et al., 2022). In their latest update, they identify that six out of the nine boundaries have already been crossed (Richardson et al., 2023). Overstepping these thresholds increases the risk of large-scale irreversible environmental changes. Taken together, the boundaries mark a critical zone in which the risks to humans and ecosystems increase as well as the levels of uncertainty. Boundaries represent interconnected processes within the complex biophysical system of the Earth.

In the crucial boundary Biosphere Integrity, Steffen et al. (2015) estimated the extent of the stress which we humans place on the biosphere integrity, differentiating between the two key aspects: *genetic diversity* and *planetary function.* The first one relates to the role of “genetically unique material” that ensures that life continues to coevolve and adapt to abrupt changes in the Earth system. The second one issues the extent to which the biosphere can fulfil functions that are important for the ecosystem services we derive, such as carbon storage or air and water filtration. The intricate web of life, from the smallest microorganism to the largest species, supports the ecosystems that sustain us all. Recognizing this interconnectedness requires a paradigm shift in our approach. It is no longer enough to pursue growth at the expense of the environment; we must strive to live in harmony with nature. This requires a fundamental rethinking of how we do things both at a personal and systemic level.

Another sign of the *Anthropocene* is the discharge of chemical pollutants, both in the form of greenhouse gases, but also in other forms, captured in the term ‘novel entities’ in the Planetary Boundaries framework (Rockström et al., 2009). Novel entities represent any synthetic material created by humans and discharged to air, water or soil with known or unknown consequences for living organisms and ecosystem integrity. One example we discuss in our study is plastic pollution, which in itself was coined as a novel entity (Villarrubia-Gómez et al., 2018) and is also associated with many other chemicals (Monclús et al., 2025). Plastic pollution is a global challenge with an estimated 23% of plastic produced ending up in the environment (IUCN, 2024). Through the action of our water bodies and weather system, plastic enters the most pristine and remote parts of our planet (e.g., Cyvin et al., 2021). Plastic decomposes slowly but surely into micro and nanoplastic and is now shown to be present in all the spheres of our planet and to have permeated in most organisms through feeding, breathing and skin contact (ref). More recently, plastics were found in human lung tissue, digestive systems, brain, reproductive organs and most shockingly in human placenta and newborn stools (Zurub et al., 2024; Zhang et al., 2021). As plastic production has reached 460 million metric tons annually (IUCN, 2024) and continues to expand unabated, plastic pollution is clogging our planet. Although research has made the link between plastic contamination and several health conditions in animal (Bucci et al., 2020) and cell damage in humans (Arranz et al., 2024) the grand experiment of plastic continues unabated, and little is known of the long-term effects it will have on ecosystem and human health.

### Shifting perspectives

The first step in shifting to a fundamental rethinking is to reframe our current understanding of what we consider as Nature. We generally consider it as a passive element placed outside of built environments, a resource that can be exploited and altered to accommodate artificial needs. Even according to the Oxford English dictionary, *Nature* is defined as the ‘phenomena of the physical world collectively, including plants, animals and other features and products of the earth itself, as opposed to human and human creations’ (Oxford English Dictionary, 2024). Clearly, nature is perceived as something in opposition to humans, and to the world of humans. However, species live interconnected with each other and with their biophysiochemical support system, always interacting. Nature is relation. Indeed, one of the earliest to advocate for wilderness preservation, the environmental philosopher John Muir expressed this notion as follows:

*‘When we try to pick out anything by itself, we find it hitched to everything else in the universe.’*

Muir (2010)

What Tsing refers to as ‘more-than-human sociality’, where social means ‘made in entangling relations with significant others’ (Tsing, 2013). Similarly, Dias draws the parallel between plants and humans by highlighting that ‘we do not think alone, but through bridges, connections, and synapses’ (Dias, 2023). Supporting the concept stated that nature should be seen as a whole, and sharing the vision of a unified nature is the naturalist Alexander von Humboldt. They both approached nature with an explorative attitude, combining direct observation with emotional and instinctive aspects, to try to understand why humans value themselves higher than everything else (Wulf, 2015).

Feelings and emotions are meaningful data to be taken in consideration when studying nature. However, this understanding of nature is not so common outside academia and the scientific realm, as humans still generally perceive nature as a passive background, giving rise to the concept of human exceptionalism, i.e., the belief that humans are the most important species on the Earth. For instance, in the Western worldview, we find the idea of the human figure as the ‘the perfect scale’ to measure and design everything; from the Vitruvian Man by Leonardo da Vinci to the Modulor by Le Corbusier and more recently in the concept of human-centered design. This vision is opposed to the one of indigenous cultures in which the human being is rarely placed over the natural world or seen as something *other* (Beery et al., 2023). One recent example is the fight of the Māori tribe to legally recognize the Whanganui River in New Zealand, granting it the same legal rights as a human being. For 140 years, the tribe has been arguing that the river should be regarded as a living entity rather than a resource that can be owned and managed (The Guardian, 2017). In her book *Braiding Sweetgrass: Indigenous Wisdom, Scientific Knowledge, and the Teachings of Plants* (Wall Kimmerer, 2020), ecologist Robin Wall Kimmerer introduced the concept of TEK - Traditional Ecological Knowledge, an approach centered in the interconnection of all living beings and their habitats that advocate for a relationship between humans and the natural world based on gratitude and reciprocity, acknowledging the role that humans play in ecosystem restoration.

### A change in ethics

**Figure 1:**
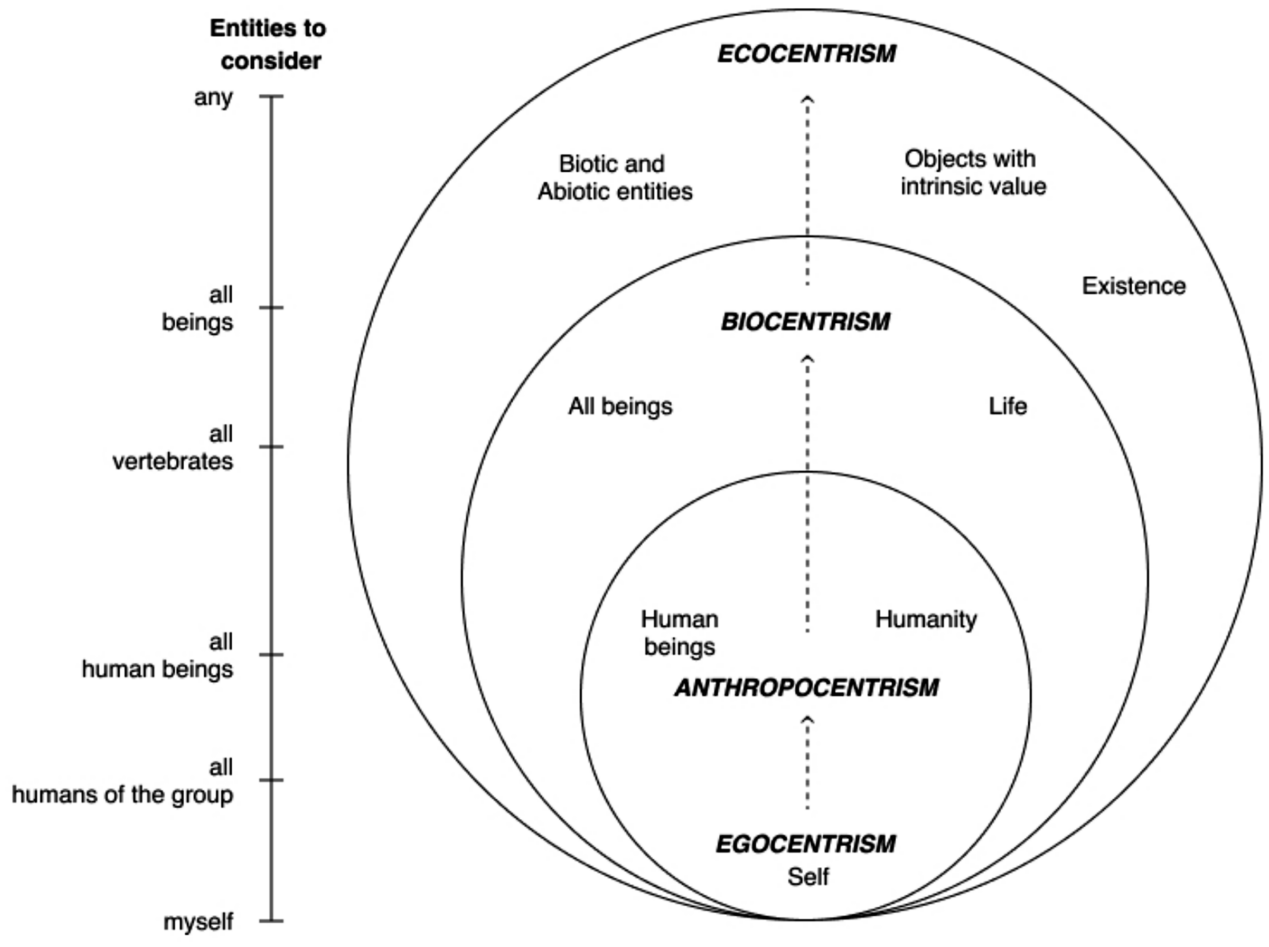
The different types of centrism and the consideration of entities and values, (scheme adapted from Rülke et al., 2020).

The concept of anthropocentrism comes back in environmental ethics; where there are three main value systems that underlie support for environmental issues: *anthropocentrism*, *biocentrism* and *ecocentrism*. All these visions express environmental concern and an interest in preserving natural resources, but their interest lies in different motivations (Samuelsson, 2013; Kopnina et al., 2018). *Anthropocentrism* is the belief that humans have a greater intrinsic value in comparison to other species (Goralnik, 2011). In that view, a healthy ecosystem is fundamental to support and benefit humans. *Biocentrism* considers that all living beings have inherent value and places greater importance on living components of the environment. It does not consider abiotic factors of the environment (Derr and McNamara, 2003). *Ecocentrism* values the ecosystem, considering equally important both biotic and abiotic components, involving also chemical and geological components of nature when supporting environmental issues. Ecocentrism considers nature worth preserving because of its intrinsic value, not for utilitarian reasons (Washington et al., 2017) (Figure 1).

Although there are differences among various anthropocentric positions, there are also commonalities that do not bode well for nonhuman well-being and biodiversity. Principal among these is the lack of ethical consideration for the intrinsic value of non-human forms. Presuming there is a difference between legitimate and illegitimate concern for human well-being leads to the assumption that humans are the only arbiters of what is “legitimate,” failing to recognize the value and right of nonhuman entities. Of course, there is the “evolutionary selfishness” to feed and reproduce and that caring for the members of one’s own species can be “good” and “natural,” but not at *the expense of* other species. Anthropocentrism is clearly a significant driver of ecocide and the environmental crisis, for society has been pursuing the project *human planet* without considering that humanity is fully dependent on nature (Kopnina et al., 2018). Ecocentrism, in contrast, accepts that we are part of nature and have a responsibility to respect the web of life and heal the damage caused by the ideological dominance of anthropocentrism (Washington et al., 2017).

In the long term, human survival is directly linked to our relationship with the fabric of life and its support system, in which everything is finely interconnected. We are all living in the same *critical zone*, as Bruno Latour calls the layer that goes a few kilometers under our feet and above our heads (Latour and Weibel, 2020). However, to truly be a ‘unified whole’ and to move away from the hierarchical perspectives of human versus non-human, we need to learn new patterns of thinking and relating that recognize that the world is not just about us.

### Venturing beyond human-centered through empathy

*Design is one of the basic characteristics of what it is to be human and an essential determinant of the quality of human life. It affects everyone in every detail of every aspect of what they do throughout each day. As such, it matters profoundly.* (Heskett, 2005, p. 2).

Changing people’s perspective and behavior in relation to the extinction of nature experience is relevant to the field of Design. Within this field, concepts such as *more-than-human* design or *post-human* design (Forlano, 2017) have gained momentum in the past years, with more research on how to ‘decentre’ design (Nicenboim et al., 2025). This field not only explores how to design space and objects to take into account the more-than-human but also uses technology to facilitate communication with the non-human (Steiner et al., 2017) and addition of body parts to explore the world from a different perspective (Giaccardi and Redström, 2020). This process, referred to as ‘embodiment’ has been shown to profoundly change the user’s connection to nature (Guy et al., 2024).

The terms *more-than-human, non-human* and *other-than-humans* refer to the concept of the coexistence with other biotic and abiotic elements alongside humans and their interconnections. A shared claim of these concepts is that humans have no intrinsic rights to prevail over any other entities, but their rights exist on a spectrum with the ones of any other species.

In her book *Thus Spoke the Plant. A Remarkable Journey of Groundbreaking Scientific Discoveries and Personal Encounters with Plants* (Gagliano, 2018), Gagliano talks about how much is necessary to “unlearn the distinctions” with which we separate us — the human — to the others — more-than-human. Unlearning the distinctions means to stop codifying the world using humans as a parameter in order to avoid considering the qualities and characteristics of *the others* as opposed or inferior to ours. She conducted an experiment (Gagliano et al., 2014) to prove that the plant *Mimosa pudica* stores and retrieves information for over a month. We are used to connecting the concept of memory to the brain, therefore we are drawn to think that since plants do not have a brain — or at least not our brain! — they cannot have the same functionalities. This experiment shows us how our convictions about ourselves, our capabilities and our being can be limiting to see the potential knowledge and information coming from the non-humans surrounding us. In this frame, disruptive eco-visualizations act as a tool to bridge the level 1 of Stein’s empathy model — Recognition and description of subject’s emotional state — with the level 2 — Relationship and comparison to a parallel experience — by breaking the language barrier issue and creating a physical body to help people to embody *the other.* It is not done by trying to adapt the knowledge, language and body of *the other* to ours, but the other way around, giving us the means to merge ourselves in the *others’ point of view,* shifting our perspective, allowing ourselves to see and feel through different eyes.

While the terms *non-human* and *other-than-human* suggest the existence of entities that are juxtaposed to the concept of humans, it amplifies the separation between *us* humans and the *others*. In our research, we choose the term *more-than-human,* coined by the ecologist Abram (1996), that does away with the hierarchy between species and gives back the right to all living and non-living entities. Moreover, it must be specified that all three abovementioned terms can also include technological entities such as cyborgs, artificial intelligence, sentient network, etc., which is reflected in our study in the form of a wearable robotic tails and eco-visualizations using machine learning. Disruptive eco-visualizations aim to question and provoke established systems by breaking into our habits and routines, shaking our social norms and beliefs to give us practitioners a way to make people temporarily forget about themselves in favor of *the others*. The differentiation between disruptive and non-disruptive eco-visualizations lies within the discussion that they spark. It is not something meant to be passively acknowledged, but rather something that stays with you and resonates with your experiences. It does that by engaging and tackling more senses and deepening the connection with them.

However, there is little research on how empathy can extend beyond humans. Schnegg and Breyer (2022) conducted multispecies ethnographic research during a 2 years period in Namibia, to explore how empathy works between humans and more-than-humans. To do so, they used Stein’s empathy model (Stein, 2012) and took in consideration the relationship between Namibian and three different others: elephants, spirits and the winds. The conclusions they draw is that (a) there is a language barrier issue that prevents people from going from stage 1 of Stein’s empathy to stage 2 (2012). This means that while we humans have different expressions (tone of voice, facial, bodily etc.) with which we express a given feeling, other beings express their feelings in different ways. So, a certain knowledge of the other’s way of expression is needed to understand their feelings; (b) the existence of a physical body is an important element to help humans relate to the others. For example, in their observation Schnegg and Breyer (2022) noticed a difficulty for humans to view the world from the point of view of the “wild wind.” People associate feelings to the wind (e.g., anger is associated with the strength with which it blows) but without the expressivity that comes with the body they fail to embody it; (c) when people do not go through the first two stages of the empathy process it is increased the difficulty to gain a new perspective on reality improved by the point of view of the *other*.

From these findings, it emerges that the processes of disconnection from nature and more-than-human entities through the loss of nature interactions could lead to further loss of literacy into other species’ needs and consequent loss of empathy, leading to further overexploitation and degradation of nature. The empathy model defined by Stein shows the role that empathy can play in bringing people closer to nature. Following this model and implementing it in a design process can eventually help to develop a framework to define better and more effective interventions to disrupt this vicious cycle.

In this study, we experimented with different methods to blur the boundaries between humans and nature, or the more-than-human. The idea that underpins these interventions, is that we need to encourage people to bond with nature more deeply and genuinely, so that this may foster empathy and meaningful reflections on our role as humans in the ecosystem. In two workshops, we used disruptive eco-visualizations (Löfström, 2017) to explore how nature impacts us as an intrinsic part of a whole, and how we impact nature through our hyper-consumerism, and how this, in turn impacts us as part of nature. In the next section, we will detail the specific methods used in both workshops.

## Methods

As our research questions concerned how to convey ecological insights in a way that also engages emotions, and as a basis to shift perspectives and behavior to ecocentric, the practical goal of the interventions were to provoke emotional resonance and empathy with nature and use this as a starting point for collective reflections and the joint co-creation of a more sustainable future. We therefore endeavor into a transformative approach. This kind of explorative studies are generally qualitative, and ours is no exception. We want to explore our topic with an open mind and learn as much as possible from the process. So, we designed two interventions, and the goal was not to compare the effects of each but rather to explore each as unique. To provide us with sufficient experiences and material we tried our concept in two workshops with different settings (urban vs. rural), audiences (age and mix of ages), timeframes and audience sizes. We used different interventions with the intent of creating ‘transformative experiences’ for the participants as part of a structured co-creative workshop method called *vision workshops*^1^ (Löfström et al., 2021; Löfström et al., 2020).

The intent with these interventions was to: (a) provoke an emotional response amongst the workshop participants and thus convey ecological insights in a manner that engages emotions, and (b) to see how, we can use these insights as a starting point for collective reflections and co-creations of a more nature-connected way of life. Both workshops were audio-documented and photographed, and one was also partly documented with video. In the first workshop, we also used a qualitative short survey, carried out pop-up interviews and asked the participants to illustrate the “re-connected man.” Our project builds further on the ideas and methodology developed in a research project (see footnote 1). Another inspiration was previous studies on technological body enhancements, and we used a mechanical artificial human tail which was originally developed for another project, which used bodily extension that brought forth intriguing bodily experiences and prompted reflections on the integrated role of technology in human experience (Svanaes and Solheim, 2016; Shusterman and Svanæs, 2023). Given the study’s exploratory nature, we carried out an inductive, reflexive thematic analysis drawing from the methods of Braun and Clarke: we first familiarized ourselves with the material, then undertook open coding, and iteratively developed, reviewed, and named themes; throughout, we kept memos and an audit trail to document decisions and enhance transparency and reproducibility (Braun and Clarke, 2006; Braun and Clarke, 2019).

We will briefly describe the two intervention concepts that inspired us in doing this study, disruptive eco-visualizations, and the ‘overview effect.’

### The disruptive eco-visualizations

*“Eco-visualization is the dynamic means of revealing the consequences of resource use in order to promote sustainable behavior, decision making and/or attitudes.”*

Löfström (2017)

The eco-visualizations used in our workshops are to different degrees designed to be ‘disruptive’, i.e., to provoke emotions. Disruptive environmental communication is an emerging field and has, so far, been used to a relatively limited extent in actual (case) studies (Klöckner and Löfström, 2022). However, the disruptive communication concept, as such, is closely related to the field of critical design, design noir, and more recently provotyping, where design with provocative elements is used to evoke discussions and reflections in a co-creative setting (Löfström and Fjællingsdal, 2022). Disruptive communication is defined as communication that disrupts everyday life (Klöckner and Löfström, 2022) in a way that makes the receiver stop and wonder about a particular topic or way of doing things that is taken for granted. In many ways, this idea is related to art, especially modern and contemporary art, whose main goals are to provoke. What follows the eco-visualization is just as important in the intervention. Indeed, the provocative element and the emotional reaction are followed by a co-creative process of “reframing” (Löfström and Fjællingsdal, 2022), where we re-build together a vision for a new future more connected to nature and more eco-centric.

### The overview effect: (technologically) enhanced ecological experiences

In addition to eco-visualization and disruptive environmental communication, a source of inspiration in conceptualizing this approach has been the ‘overview effect’, commonly reported by astronauts viewing Earth from space. This effect altered their perception of their place within the universe, and really made humanity realize that the Earth’s resources were limited. Our long-term endeavor is to simulate this transformative effect through technological and non-technological means, translating the deep-seated realization that comes from viewing Earth from a vantage point in space to experiences here on Earth. We explore the potential of various technologies to induce similar experiences, setting the stage for deeper emotional engagement. We contemplate engagements with nature, where the familiar becomes unfamiliar, invoking a heightened awareness of our inherent eco-connectedness. We hypothesize that generating an acute awareness of ‘missing nature’ through controlled environments could facilitate reflections on our symbiotic relationship with nature. In essence, we use the contrast of space vs. earth and nature vs. absence of nature.

We carried out two explorative vision workshops with different stakeholders: one in a rural and one in an urban one. At these workshops, we utilized both high-tech and no-tech eco-visualization to induce bodily experiences, as we believe this can cater to a wide audience, with different preferences and sensitivities, and can possibly create a rich tapestry of experiences that resonate with different individuals and their value systems, on different levels. The juxtaposition of high and no-tech experiences also poses an interesting dimension of exploring the human-nature relationship from different angles. The originality of our approach consists in the focus on bodily and emotional experiences, rather than cognitive ones as we wished to explore the effects of emotions on reconnecting with nature.

### The first workshop

Our first workshop was set to a remote island in the Danish archipelago, Samsø^2^. After an initial presentation on the intent of the workshop, the participants were exposed to our two interventions in this rural setting; (1) sending the participants out on a silent individual nature-walk with no technology, and (2) offering the participants (on a voluntary basis) to try wearing the robotic tail that will move with the person wearing it. During the workshop, the participants were asked to discuss in plenum, and in pairs, and they made an illustration of “the eco-connected (hu)man” on a paper with a simple dash man as starting point.

For the first intervention, the silent individual nature-walk, our instructions were simple and in the line of: Take a silent walk in nature. Use your senses, feel the wind, smell the ground and touch the grass. Allow yourself to be silent. For the second intervention, we had brought five robotic tails to the workshop, allowing the participants to test wearing them. The tail is approximately 80 cm long, covered with fur, and is strapped to the wearer’s waist with a belt. It contains sensors, motors and electronics that makes it wag in a natural manner as the wearer moves. Thus, the mechanical tail becomes an extension of the wearer, moving in sync with the wearer. For the tail experience, our instructions included a brief practical/technical instruction of how to best manage wearing the tail together with encouraging the participant to explore the *experience* of having a tail, simply doing what feels right in the moment. The rationale behind this mix of experiences – the technology intense robotic tail and the individual strictly non-tech nature experience – was that it introduced a multifaceted layer to the workshop, interweaving natural elements with technology.

The provocative elements in the first workshop can be identified with the association of the silent individual nature-walk with the act of wearing a robotic tail. They first had the chance to let go of themselves and sharpen their senses to then immerse themselves in the experience of having a tail, having a moment to linger on what emotions and thoughts this elicited. It is a shift in perspective, a chance to merge into something that is not *us.* The provocative element in the robotic tail intervention was to highlight experientially the animal side of the wearer, to depart from our duality and tendency to contrast humans with nature (Tsing, 2013). The transformative experience in this case consisted in shifting perspective from being detached from nature to being embedded in it with sharpened senses and awakening to the present moment.

The participants of the workshop were all attending the Samsø annual festival “Foooolkedybet” organized by the Energy Academy^3^, and our workshop was part of the festival program. We carried out convenience sampling as the participants were all voluntarily attending the workshop based on the description we had provided in the program. The total number of participants of our workshop was 24 persons, with ages ranging from 22 to 74 years of age. Most of the participants were residents of Samsø, while a few were land-based visitors who were there solely for the festival. After the workshop ended, we carried out brief pop-up interviews with participants and handed out a short survey to collect feedback on the workshop.

### The second workshop

**Figure 2:**
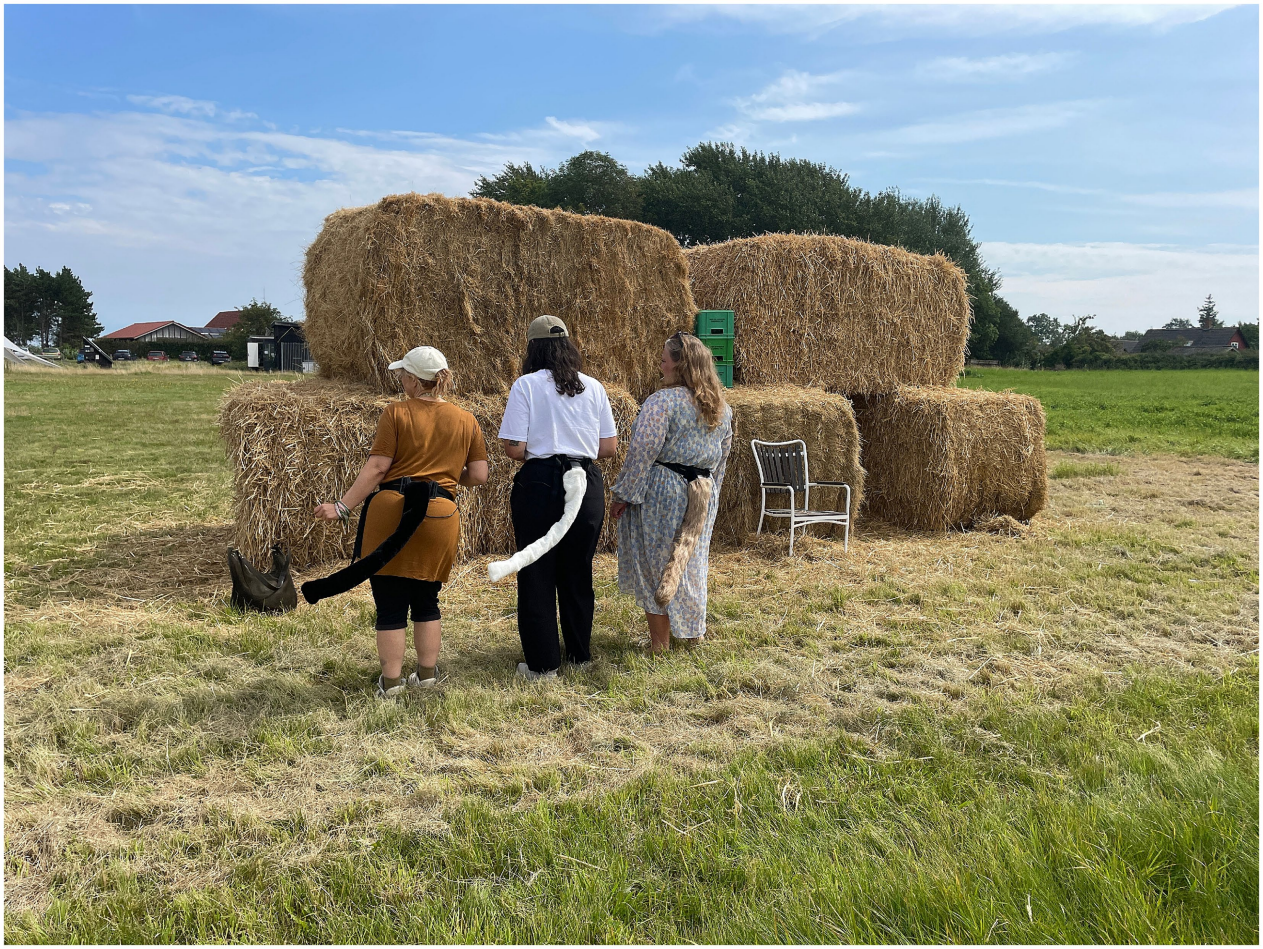
Participants testing the robotic tails the picturesque landscape of Samsø Energy Academy, here “wiggling their tails” as part of the workshop.

**Figure 3:**
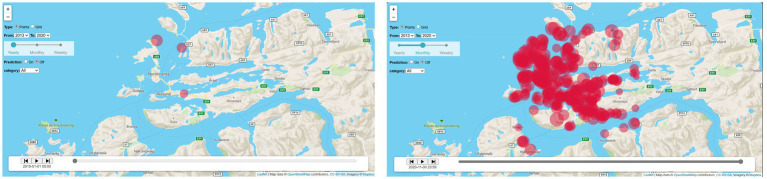
Screenshot of the visualization tool from the PlastOPol marine plastic pollution data platform: left panel shows conditions in 2015; right panel shows conditions in 2020 (Wu et al., 2023).

The second workshop was carried out in an urban setting, as part of the youth program of the largest political gathering in Norway held annually, namely ArendalsUka^4^. Our workshop initiative engaged a hundred adolescents in the age bracket of 16 to 17. Here again, we carried out convenience sampling, as we designed the workshop program together with GridArendal, who then invited local High Schools to take part on one of the days of ArendalsUka. We had designed an educational experience to foster a deeper understanding of environmental issues, particularly focusing on the proliferation of plastic waste in marine and coastal ecosystems. The workshop consisted of a short introduction which included a dynamic eco-visualization illustrating the spread of plastic in natural environments, offering the attendees a tangible representation of environmental degradation (see Figure 2). The visualization is part of a data system developed through a regional project and shows the findings of plastic around the Norwegian coast of Møre and Romsdal as dots of various shades of red, depending on the amounts found, over a timeline between 2013 and 2020 (Wu et al., 2023). This visualization shows the booming increases in plastic found along this relatively low-populated and remote area of Europe, and also the increase in collection activities (Figure 2). The provocative element in this eco-visualization was the graphics of the datapoints and their accumulation over time, which made the plastic spread as a disease similar to chicken pox. The eco-visualization was used with the goal of visualizing an ‘overview effect’ of the plastic pollution found along the coast of Norway. The aim was to take a step back and truly grasp the magnitude and cumulative nature of the issue. This in turn, we hypothesized could provoke a shift in the pupils’ experience of plastic pollution going beyond the occasional item they see on their visit to the coast, to cumulative over space and time and the vulnerability of nature and its limited capacity as a sink. The participants used plastic as a way to reflect on the meaning of the impact of our actions and decisions on our surroundings (Figure 3).

After the workshop introduction, the adolescents were divided into 10 randomized groups and presented with a long table with plastic waste. Glue, pens and white A3 sheets were also available. We encouraged the groups to create art that would symbolise human nature. We also presented them with the following discussion points in relation to the recovered plastic that they were encouraged to discuss while co-creating their artworks:

- How and why did this end up in nature?
- What can we do to avoid this?
- How does it feel?
- Did we really need this?

The workshop was enriched with an element of playfulness through facilitators sporting robotic tails, aimed, in this case, at breaking the ice and establishing a congenial atmosphere for collaborative creativity. We had brought the same robotic tails that we had used in our rural workshop on Samsø, but the participants were too many to allow all to test it. Therefore, we ourselves and a few of the facilitators wore the tails during the full workshop, including while walking around interacting with participants. In addition to the project researchers, we had access to facilitators affiliated with Grid Arendal^5^, and the accompanying educational personnel from the pupils’ respective schools. These individuals gently guided the groups in a collaborative art project, encouraging them to craft artworks utilizing the plastic materials. As we were interested in what the participants were going to make of the task – we did not want to limit them – we instructed the facilitators to be supportive and contribute to a safe environment. Therefore, the facilitators were to have a “yes-and” approach to the task and to all the participants´ suggestions.

The artistic endeavor was more than a mere creative exercise; it was envisioned as a starting point for a reflective discourse on the intricate relationship between humans and the ecosystem. It encouraged the participants to ponder on the ramifications of environmental degradation, instilling in them a heightened awareness and fostering a sense of responsibility towards nurturing a sustainable relationship with nature. By melding visual narrative techniques with a hands-on artistic experience, the workshop sought to spark a conscientious dialogue about environmental stewardship among the younger generation.

At the end of the workshop, all groups presented their artwork in a plenary session. Most groups had worked as a team to tackle the challenge, but there were two cases where pupils had withdrawn from the group and instead made their own artwork. We did not try to change this but let these pupils work on their own.

## Results

### The first workshop

The response to the “being alone and silent in nature” – exercise rendered strong responses. When returning from the silence, people were initially reluctant to speak. It was as if they were all suddenly in listening mode. This was surprising as the overall energy level had been quite chatty before this exercise. When starting to discuss in groups of two, we could notice two different kinds of responses from the participants. Some of the locals shared previous experiences of being alone in nature, and referred to a feeling of completeness:

*I often take silent walks in nature. Your senses kind of get sharpened. I think it’s because we are so in nature all the time. It kind of holds us*.

Local woman, age 56.

*I believe we humans can be so much more, actually. We could live in sync with nature. But first we need let go of all the things we think we need. We need to become nature.*

Local woman, age 39.

For visitors of Samsø, this experience was both frightening and fascinating. It appears the experience had added a level of vulnerability to *being human*:

*I felt as though my senses were sharpened. As if the grass was gnawing on my bones. It’s kind of scary suddenly feeling so small.*

Visiting man, age 63.

*I had a feeling of not belonging. Like I had crashed a party. It’s probably because we are the species of destruction*.

Visiting man, age 35.

All in all, this non-technological experience was surprisingly transformative, and the workshop continued with a sense of calm and thoughtfulness. This far exceeded our expectations and we took part of many interesting discussions with participants after the exercise was completed. After approximately 40 min we switched to the technological robotic tail experience. The five tails we had brought was not enough to allow everyone to test it at the same time. Also, testing it was of course on a voluntary basis. Surprisingly, only two persons volunteered to try it on immediately. Possibly, the initial non-technological exercise had resulted in some form of shyness amongst participants. However, once a couple of persons had tried the tail on for a while, others were also willing to try. Quite opposite to the non-technological experience, the robotic tail seemed to energize those who wore it. People started to jump around while wagging their tail and participants with tails started experimenting with communicating using solely the tails. Funnily, a dog that belonged to another festival visitor came up to one of the tail-wearing participants and started barking at her. The experience was playful and opened for discussions of what could be:

*I wonder what I said to him? Perhaps I was rude. It’s not like I have learned to speak tail… Perhaps I should, we all should.*

Local woman, age 52.

*I felt like I was returning to the very beginning of mankind. Like Eve before the apple, kind of. What if we could start anew and do things right this time?*

Visiting man, age 36.

At the end of the workshop, we had short talks with participants and asked them to reflect. It seemed that taking the tail off had resulted in a feeling of “taillessness” and that the overall feeling remaining from the workshop was that the participants felt they had been “shaken in their core.” This made the wearer reflect on the role of technology in our human lives. Some participants reported wishing or longing for a simpler life, with fewer industrial products and services. During these reflections, we noticed that the participants were starting to venture out of the anthropocentric perspective of nature and into the so called more-than human realm (Abram, 1996):

*We do not have nature; we do not own it. But we treat it like a great endless buffet that we can just pick and choose as we please from*.

Local woman, age 52.

This reminded us of mythological dreams of unlimited access to food and drinks. For instance, in the Viking version of Afterlife, Valhalla, Sæhrímnir, is a boar that is cooked every night, as he is the never-ending food source for the valiant warriors. In the case of the tail intervention, we did not see the same divide between local and visiting workshop participants.

### The second workshop

In our thematic analysis, we identified three clear themes which the groups’ artworks–and particularly their own descriptions of these artworks–fit into; namely Making Nature Artificial, Humans’ Insatiable Greed and Abandoning Earth.

### Making nature artificial

The dominating theme of artworks that were developed in the groups can be categorized as what we call the “Making Nature Artificial”–category. We had four group artworks belonging to this category, and two pupils who worked alone. The variations of these artworks were relatively broad, but they all had one thing in common, namely, they used artificial materials to represent nature in their artworks. A few groups and individuals made plastic flowers and gardens imitating nature, and this was to a certain degree expected. After all, we had provided them with plastic and asked them to create. However, although these artworks were to different degrees aesthetically appealing, it seemed that all youngsters who had created them had used the task as a starting point for reflections on our current and potential future role as humans in the ecosystem. Below are some of the reflections that were made by the creators of these artworks:

*“I believe we are destroying nature and then trying to replace it. We can make it look like nature, but it’s not alive.”*

Girl working alone

“We do not want nature to be wild, do we? We want it to be pleasant and beautiful. I think it’s beautiful even when it’s wild.”

Girl in group

**Figure 4:**
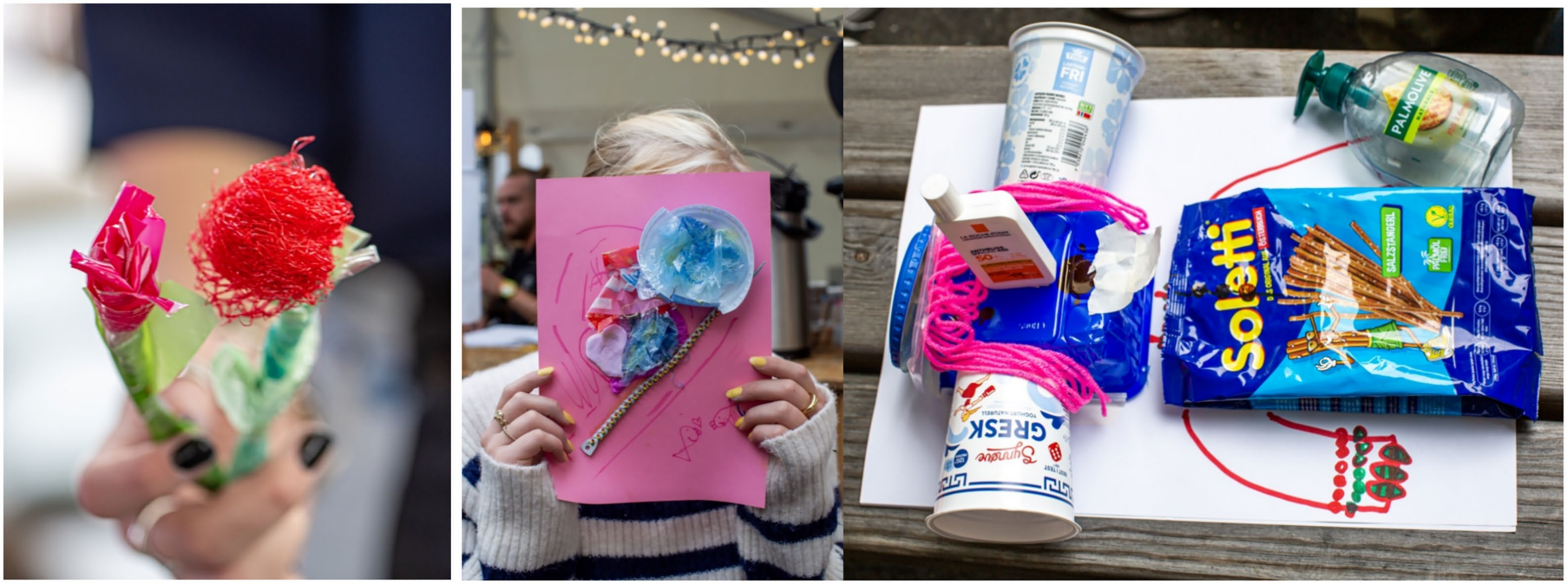
Photos representing (from left to right) flower by a girl working alone, marine ecosystem by a group and a plastic robot man by a group. Adapted with permission from GRID-Arendal, 2024.

Other groups made artificial beings that were to different degrees still human or animals but had become artificial or plasticized. These groups all referred to the occurrence of microplastic in nature, and how it spreads without us being able to detect it (Figure 4). To them, microplastics seemed to represent the invisibility of climate change and other environmental issues, and they used this as an outlet for their worry for the future of mankind:

*“This used to be a man, but now it is this creature. He is like a robot because he has become the plastic he consumes.”*

Boy in group

### Humans’ insatiable greed

The second largest category of artworks amongst the groups, we have chosen to call Humans’ Insatiable Greed. This category fathoms artworks with a relatively dark symbolism, namely that we humans as a species seem to have an insatiable greed, and that we are inherently incapable of stopping ourselves. We keep consuming and destroying the planet in the process. The descriptions and reflections of the groups whose artworks belong to this category all boil down to that: we do not stop (consuming/destroying the planet), we will not, because we cannot:

*“We have recreated a glass elevator building here in Arendal. It is huge, but we have made it even higher here, we added another layer. It’s because we always must make everything bigger if we can.”*

Boy in group

This elevator was also fully functioning and had movable parts with a weight that could raise the elevator floor up so you could see it through the “glass” walls of this high tower (Figure 2). We got to think of both the Tower of Babylon from the Bible where man builds a tower rising high up to the heavens, and the myth of Icarus from Greek mythology, who perished by flying too near the Sun with waxen wings.

**Figure 5:**
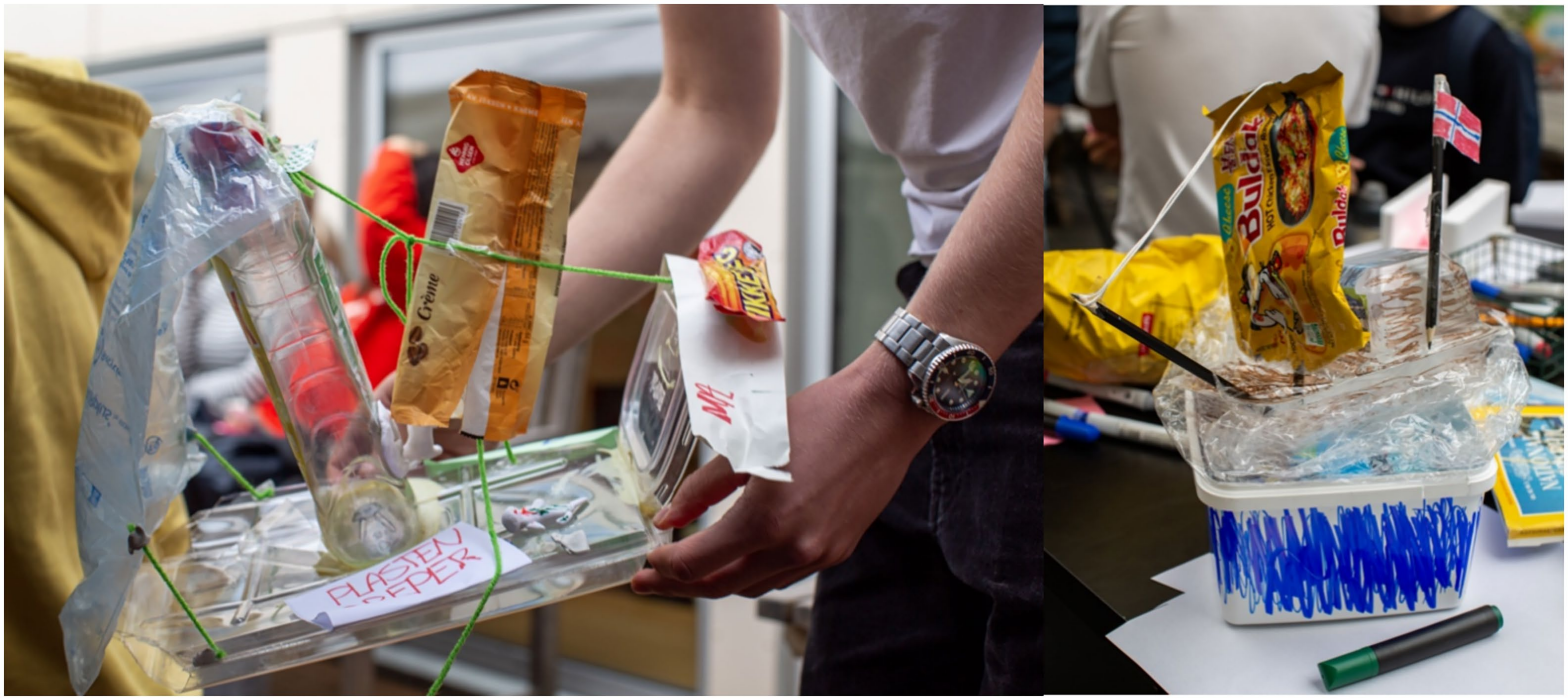
Artwork showing from left to right an elevator and a boat. Adapted with permission from GRID-Arendal, 2024.

The two other groups with artworks in this category had both made boats that were sailing on the ocean steamed by human consumption or greed (Figure 2). One was more focused on plastic and made a point of that humanity had – in a not-so-distant future – created boats that were entirely made of plastic and kept getting bigger and faster by our inability to change our ways (Figure 5). The other group took it a step further and described the extinction of humans due to our “insatiable greed”:

*“It runs on our greed. Our species has this insatiable appetite that consumes everything in our path, and we cannot stop. We just keep consuming.”*

Boy in group

All the groups in this category seemed to be using a dark sense of humor when talking about their artworks. According to Martin (2007, p.350) humor can be useful in teaching ‘sensitive, anxiety-arousing topics’ and Banas et al. (2011) have concluded that it may relieve tension when the subject matter is anxiety-provoking. In our study it seems that some youth use this intuitively as a means to tackle their climate-anxiety. Also, all three groups used extensive body language to illustrate how greedy and needy we humans really are, making their talks with us into a form of ironic pantomime.

These two boats artworks again made us think of myths and folklore. In ancient times, when people believed the Earth was flat, seafarers are said to have been afraid that their ship might fall off the edge of the world. To us, while listening to the creators of the artworks, falling off the edge of the ocean did not seem unlikely for these two ships.

### Abandoning earth– humans saving themselves

The third and smallest category was that of humans abandoning ‘ship’. This artwork was described as a spaceship heading for other habitable planets. Only 50 humans would fit on each ship, and they were to form colonies on other planets where humanity could start anew. This group also used their sense of humor when referring to their artwork. Again, we interpreted this as being their way of handling the grim view on humanity that they were proposing through their artwork narrative:

*Universe, beware! Here come the worst vermin. We are deadly!*

Boy in group

## Discussion

Interestingly, in the first workshop, there appeared to be a division between the participants who were visiting the island from urban areas, and those who were local to the nature-rich island. When asked to walk in silence, the locals seemed to feel at home in nature and to realize that it was not just there to be exploited. On the other hand, some of the visitors to the island expressed fear and a feeling of not belonging or ‘gate-crashing’. This could be easily explained as the nature surrounding the latter was unfamiliar to them. However, it could also confirm a previously discussed theory that urban dwellers are more disconnected from nature as they do not experience it to the same degree or as often leading to a form of uneasiness (Soga and Gaston, 2016).

The intervention of wearing robotic tails had some surprising effects, as the participants in the workshop got used to wearing them very quickly and taking them off seemed to provoke a feeling of loss or ‘taillessness’. The remark the participant made on communicating with a dog points to the language barrier highlighted by Schnegg and Breyer (2022) as an obstacle to developing empathy. The dog’s barking made the participant humorously remark that she did not know ‘tail language’ and that she might have been unintentionally rude. Although it is common knowledge that animals use their tails as a way of expressing emotions, this form of experiential learning, where one provokes a reaction through miscommunication seems more powerful.

Overall, the tail experience appeared to open for becoming an animal, becoming *more nature*. The tail seemed to work as a mental re-connect with nature. Through the walk in silence, the participants first successfully connected to nature and became more aware, more listening. Then, the use of technology opened for playfulness. After taking the tail off, however, a feeling of amputation was sparked, taking something away, namely a ‘body-part’ that had previously been accepted by the body as an extension of itself. This is a practice used in more-than-human design through the process of embodiment, thereby blurring the boundaries between their bodies and nature through their senses.

Whilst in the first workshop, we blurred the boundary between human and nature by making participants *more nature;* in the second workshop, we highlighted humans’ impacts by making nature *more human* through plastic permeating through all the Earth’s systems and accumulating. The pupils picked up on this idea and reflected it in their artworks and explanations. Interestingly, one of the groups, who created a robot/plastic man, highlighted the fact that what we do to nature comes back to affect us as being part of nature, completing a full cycle. This is a deep and insightful thought that goes beyond the mainstream narrative of plastic pollution as being mainly an environmental problem. It points to the fact that, as we depend on nature more than we realize, the novel entities we impose into wild organisms and their abiotic support system also permeates back into our fragile and interconnected body systems.

Many of the groups used their sense of humor both when choosing the design of the artwork and when commenting on it. We interpreted this as a coping mechanism to the stress caused by the task and by their vision of the future, which seemed to be perceived as both scary and inevitable. We must recognize that adolescents may have a need to be funny when talking with and in front of their classmates. Moreover, humor could in this case be a form of resistance to perceived adult inaction on the problem. However, humor and irony have been previously identified as a coping mechanism both in school contexts to protect the self in cases of tension (Woods, 1983; Banas et al., 2011; Martin, 2007), and in other contexts, such as health (Kuiper et al., 1993). Only recently is humor being investigated as a learning tool for climate change communication (Boykoff and Osnes, 2019; Carroll-Monteil, 2023). However, apart from it being mentioned as a possible coping mechanism for climate and eco-anxiety in recent Master’s theses (see for instance Väänänen, 2025), it is not explored in peer-reviewed literature. We believe this is an avenue for further investigation.

A common theme in both workshops is that of fear. In the first, it was hinted that nature could be frightening; whilst in the second, it was the plastic-clogged future that was daunting. The first form of fear can be identified as biophobia (Soga and Evans, 2024). In their description of biophobia as a vicious cycle, Soga et al. (2023) explain how disconnection from nature leads to more biophobia which in turn leads to more removal of natural areas or individual species in a positive feedback loop. Biophobia or ecophobia is proposed as the emotion felt by colonizers who destroyed native forests and tamed ecosystems (Delaney, 2024), as described in Robinson Crusoe (Defoe, 1719) and Conrad’s Heart of Darkness (Conrad, 1899). It is also believed to be on the rise in an increasingly urbanized world (Soga and Evans, 2024).

This points to the need for more such interventions to reconnect with nature and cultivate a pro-environmental ethos. Not only on a personal level but on a global level. Indeed, nature connectedness is considered as a leverage point for sustainability, as nurturing connections with nature at specific places in a complex system can have far-reaching positive impacts (Abson et al., 2017). This may also impact how future generations relate to nature.

The second form of fear of a polluted future is recognized as eco-anxiety. This form of anxiety is an extension of climate anxiety, which affects particularly young and vulnerable people and is characterized by fear related to the ecological and climate crises (Coffey et al., 2021). It is on the rise due to the continued environmental degradation and the lack of political action. Eco-anxiety becomes problematic when it impairs the normal psychological development of children and young adults and when it causes depression (O’Hare, 2022). Although humor may help overcome this anxiety temporarily or in public, it is unclear how we can encourage young people to reconnect with nature, when their perception of it is that it is damaged beyond repair and that humans are on a hopeless path. This is alarming, but not surprising, as it confirms the results of the Hickman-survey (Hickman et al., 2021). However, we argue that we should not address this eco-anxiety solely as a problem *per se*, but rather address the problem of ‘code red’ for humanity in a meaningful way, engaging young generations as well but also delivering our part. After all, it is rational to be troubled about the state of the world today.

Through our second workshop, we attempted to spark a transformative experience from waste to artwork, by encouraging pupils to craft their own stories and artefacts, and reflect, while doing so, on how to limit human-induced environmental damage and reconnect with nature. This follows best practice in Environmental Communication to avoid only burdening the receiver with information that will produce a negative stimulus, but to complement information with activities that target solutions and hope, thereby stimulating the feeling of self-efficacy in changing behavior (Klöckner, 2015). The next step would be to direct these solutions to the more structural and systemic changes that the state of the environment requires and to avoid the feeling of hopelessness that the ‘overview effect’ can create, namely to expand the effects of the behavior change to a larger form of collective or systemic change.

It seems more urgent than ever to, firstly, encourage young people to experience nature directly and to feel its calming effect. Secondly, placing too much pressure on young people to “fix” all the environmental problems seems at the very least unfair and not constructive. Young people learn about environmental challenges from a very young age and are encouraged throughout schooling to develop a stewardship towards our planet. The contrast of those ideals with the real world and the repeated observed inaction can trigger a sense of disempowerment and hopelessness. It is crucial that the education provided focuses on not only the small actions the pupils can take to make a difference, following the indications of countless studies (Klöckner, 2015), but also on the power they have in demanding wider collective, structural and systemic change. Thirdly, to attempt to address the growing generation conflict due to disappointment and growing awareness of the challenges ahead, it is urgent that decision-makers at all levels of our globalized failed system act in accordance with the scientific evidence.

### Limitations

Although both workshops used transformative experiences to spur reflections on our role in the ecosystem, much else was different in the two workshops: the setting (rural vs. urban), number of participants (24 vs. 100) and age of participants (adults vs. youth) to mention a few. These variations make it difficult to make comparisons between the workshops, and this is partly why we have chosen an explorative approach. Another aspect where we wish to, in future studies, work more systematically, is to not only include reflections and discussions on the participants’ individual and collective behavior and reconnection to nature, but we wish to add tools to stimulate discussions on systemic changes that can be activated by citizens, and the means through which they can effectuate these. Indeed, we firmly believe that it is crucial to shift the discourse from merely individual consumer choices to systemic barriers to positive changes, and research questions and practices should follow this shift. As an example, the use of disruptive communication in the form of provotypes is currently being explored as a means to involve stakeholders in co-creating new models of democracy in an ongoing EU-project INCITE-DEM, Inclusive Citizenship in a World in Transformation: Co-Designing for Democracy^6^ (Löfström et al., 2026).

## Conclusion

Our ambition for carrying out these two workshops was to, if possible, create a Proof of Concept (PoC) that our approach deserves further studies, and to learn as much as possible from the process on how to convey ecological insights in a manner that resonates not merely with the cognitive faculties but engages emotions; and use these insights as a starting point for collective reflections and co-creation of a more nature-connected life. To achieve this, we used transformative experiences as interventions to provoke an emotional response amongst workshop participants. We followed more-than-human design approaches by using technology that is tangible and visible to act as a catalyst for awareness or awakening to the impact humans have on the environment and for a reconnection with nature.

Based on our initial studies, we propose that it is fertile to continue with this approach that leverages bodily experiences of nature to explore and challenge conventional understandings of what it means to be human. Central to our hypothesis is the potential transformative power of direct ecological experiences – sometimes enhanced through technology – to foster a deeper emotional connection with the environment. Drawing on these initial experiences, we posit that the imperative of the present moment is not simply an augmentation of knowledge, but a facilitation of collective emotional awareness regarding the gravity of the prevailing ecological crisis and a practical experience of reconnecting to nature and what that would feel like.

Additionally, we wish to explore further the ideas of more-than-human design to reconnect humans to nature through physical and emotional experience. We realize that it is a long-term academic endeavor to embark on this exploratory journey. Building on this, we propose a Human–Nature Lab and Research Centre to challenge human–nature relationships through both high-tech and low/no-tech approaches—combining AI, digital maps and GIS, and AR/VR with embodied practices, analog artifacts, co-design, and futuring. The center will design, test, and scale interventions, piloting in schools, university museums, and municipalities with longitudinal studies to measure real-world impact. The goal is to also feed the results back into teaching in both More-than-Human Design and Environmental Communication, on how these new methods can help shift the focus from individual behavior change to collective, structural and systemic demands for change.

## Data Availability

The raw data supporting the conclusions of this article will be made available by the authors, without undue reservation.

## Footnotes

^1^ www.niyf.no

^2^ https://www.visitsamsoe.dk/en/

^3^ https://energiakademiet.dk/folkedybet/

^4^ https://www.arendalsuka.no

^5^ https://www.grida.no

^6^ https://incite-dem.eu
